# Healthcare provider person-centred practice: relationships between prerequisites, care environment and care processes using structural equation modelling

**DOI:** 10.1186/s12913-022-07917-3

**Published:** 2022-04-29

**Authors:** Nur Zahirah Balqis-Ali, Pui San Saw, Jailani Anis-Syakira, Weng Hong Fun, Sondi Sararaks, Shaun Wen Huey Lee, Mokhtar Abdullah

**Affiliations:** 1 Centre for Health Outcomes Research, Institute for Health Systems Research, National Institutes of Health, Ministry of Health Malaysia, Block B2, No. 1, Jalan Setia Murni U13/52, Seksyen U13 Bandar Setia Alam, 40170 Shah Alam, Selangor Malaysia; 2 School of Pharmacy, Monash University Malaysia, Jalan Lagoon Selatan, 47500 Bandar Sunway, Selangor Malaysia; 3 Jeffrey Cheah School of Medicine and Health Sciences, Monash University Malaysia, Jalan Lagoon Selatan, 47500 Bandar Sunway, Selangor Malaysia; 4 School of Pharmacy, Taylor’s University Lakeside Campus, Jalan Taylors, 47500 Subang Jaya, Selangor Malaysia; 5 MERITUS University, 49, The Boulevard, Mid Valley City, Lingkaran Syed Putra, 59200 Kuala Lumpur, Malaysia

**Keywords:** Person-centred, Primary care, Healthcare provider, Person-centred practice inventory-staff, Structural equation modelling

## Abstract

**Background:**

The translation of person-centred care concepts into practice requires fulfilment of necessary components, including person-centred values and practice held by the employees and having a supportive system. The objectives of this study were multifold: firstly, to evaluate the measurement model, secondly, to examine the roles of prerequisite or attributes of healthcare providers and care environment and how they affect delivery of person-centred processes; and finally, to examine the mediating effect of care environment towards the relationship between prerequisite and care processes.

**Methods:**

A cross sectional study was conducted among healthcare providers working in primary care facilities in a state in Malaysia. The Person-centred Practice Inventory-Staff instrument (PCPI-S) was distributed and completed by respondents. The instrument structure, reliability and validity were assessed through confirmatory factor analysis, while the framework’s unidirectional hypothesis and the mediation path hypothesis were analysed using structural equation modelling.

**Results:**

The overall goodness of fit verifies the original Person-centred Practice Framework, allowing some correlation errors. There were significant relationships between prerequisites of healthcare providers and care environment (β = 0.826, *p* < 0.001), as well as between care environment and care processes (β = 0.785, *p* < 0.001). This analysis also proved that care environment plays a partial mediating role in the relationship between prerequisites and care processes.

**Conclusions:**

In order to successfully move towards delivering person-centred practice, it is imperative to equip healthcare providers with person-centred values and beliefs, while at the same time transform current work culture to align with person-centred care. This will allow successful delivery of person-centred processes.

**Trial registration:**

NMRR-18-309-40,447.

**Supplementary Information:**

The online version contains supplementary material available at 10.1186/s12913-022-07917-3.

## Background

Healthcare systems worldwide are investing in effective approach to enhance care experience and quality care. Efforts are underway to address imbalanced and fragmented care, shifting care from medically-dominated and disease-orientated towards one that is relationship-focused, collaborative, and comprehensive [1–3]. This long-standing movement of ‘what a person requires’ was defined by Kitwood’s person-centredness as ‘a standing or status that is bestowed upon one human being by other, in the context of relationship and social being, implying recognition, respect and trust’ [4]. McCormack’s four concepts was elaborated in person-centred practice as being in relation, social world, place and self [5]. These concepts defined person-centred care to be ‘an approach to practice that is established through the formation and fostering of therapeutic relationships between all care providers, service users, and others significant to them, underpinned by values of respect for persons, individual right to self-determination, mutual respect, and understanding’ [6].

Respecting individuals as persons and acknowledging their right to care partnership appears to be consistently mentioned concepts across various person-centred definitions, as does the formation of healthy relationships between parties involved [7]. Nolan and colleagues argued the focus should also shift from trying to meet individual needs alone, to interacting with all parties involved in care whose needs should be taken account of [8]. Lastly, it is also often confused as to ‘who’ the person should be. McCormack and McCance clarified that ‘person’ refers to all involved in a caring interaction and therefore encompasses service users, families/carers, healthcare providers, and other members of the multidisciplinary team [1].

There are many benefits of person-centred practice. For example, person centred practices can help foster healthy relationships, improve self-management support and shared decision making. These all lead to an increase in patient satisfaction, self-efficacy and empowerment, leading to a change in self-care behaviours including better medication and treatment adherence [9–12]. In addition, through indirect relationship, person-centred practice also improves health-related quality of life indicators such as physical functioning [13, 14], and better healthcare utilisation such as reduction in emergency cases, hospitalisations and cost of utilisations [10, 15, 16].

In Malaysia, primary care services are provided both by the public and private sector. The public sector, which has an extensive network of health clinics, community and mobile clinics, are spread throughout the country and provides almost two-thirds of outpatient care. While recognising this access to primary care settings, prevalent non-communicable diseases, growing public expectations and ambitious health goals continued to highlight the pressing need for action on quality of care [17]. The call to reform healthcare system towards person-centredness was mentioned formerly [18–20]. At primary care level, the Family Doctor Concept initiated in 2014 was aimed to empanel the population to dedicated health teams, ensuring continuity and coordination of care [21]. The Enhanced Primary Healthcare programme in 2018 introduced screening, early illness detection, effective referral and multi-disciplinary teamwork to achieve an integrated network of person-centred care services [22]. Studies that examined delivery of person-centred care included aspects of unvoiced care needs among service users [23], and areas for improvements in shared decision making [24–26].

Reformation allows health care providers and organizations to adopt a person-centred culture to reciprocate to service users who will then benefit from person-centred health outcomes [27]. However, the challenge remains in effectively translating the principles of person-centredness into practice. Developing person-centred culture requires a sustained commitment across organisations to facilitate changes.

### Person-centred practice framework

The Person-centred Practice Framework was originally developed by McCormack and McCance [6] from studies of person-centred practice with older people [28] and nursing care [29]. The layers of the framework were ordered such that the outer constructs were to be fulfilled to strengthen the next inner layer. For example, the attributes of healthcare providers (prerequisite) must first be considered and enhanced to form a person-centred care environment. This ordering ultimately allows attainment of the person-centred outcomes; the central component of the framework [6]. The four constructs in the framework are elaborated in Table 1.

**Table 1:** Person-centred Practice Framework’s elaboration

Prerequisites	Attributes of healthcare providers and include: being professionally competent, having developed interpersonal skills, being committed to the job, being able to demonstrate clarity of beliefs and values, and knowing self
The care environment	The context in which care is delivered and includes: appropriate skill mix, systems that facilitate shared decision making, effective staff relationships, organisational systems that are supportive, the sharing of power, the potential for innovation and risk taking, and the physical environment
Person-centred processes	Care delivery through various activities, including: working with patient’s beliefs and values, engagement, having sympathetic presence, sharing decision making, and providing holistic care
Person-centred outcomes	Achieved as a consequence of effective, person-centred care and include: satisfaction with care, involvement in care, feeling of wellbeing, and creating a therapeutic environment

The framework was structured based on the Donabedian model for evaluating quality of health care defined by a triad of structure, process and outcome [30]. In the framework, the structure construct from the Donabedian model was divided into 2: Prerequisite and Care environment with the former seemingly influencing the latter [6]. The interaction between individual characteristics and organizational system is shown to be two-way and dynamic [31]. Extensive literature has discussed the importance of individual and inter-personal characteristics in shaping the organizational or workplace culture [25, 30, 31]. Various factors have been considered to define what constitute organizational culture which include customary dress, language, behaviour, beliefs, values, assumptions, symbol of authority, ceremonies and rituals, and modes of deference and subversion practiced within the organization [32, 33].

The framework comprehensively developed a solid evidence base for person-centred practice due to strong development process based on long-term and multiple studies. The framework forms the modelling base for organisational-wide strategic plan development, guides person-centred practice implementation, and utilised for evaluation purposes [7, 28, 29].

### Person-centred practice inventory-staff (PCPI-S) instrument

The 59-item Person-centred Practice Inventory-Staff (PCPI-S) Instrument was built upon the Person-centred Practice Framework and consists of 17 constructs [34]. Responses were captured using a 5-point Likert scale ranging from strongly disagree to strongly agree. The instrument was validated among 703 nurses working in eight acute hospital settings [34]. A Norwegian study utilising the translated instrument (*n* = 258) found that 6 out of the 59 items failed to achieve acceptable loadings (0.35) although 13 out of the 17 constructs had acceptable Cronbach’s alpha score (> 0.6). Another study conducted in Germany found 4 items failed to achieve acceptable factor loadings (0.6) while the internal consistency was found to be high (Cronbach’s alpha > 0.9) [35]. The confirmatory factor analysis (CFA) of the studies indicated the framework had acceptable goodness of fit [34–36]. Our first part of validating the instrument found the internal consistency to be high (Cronbach’s alpha > 0.9), while the exploratory factor analysis found differing work culture and interpretation of person-centred practice led to formation of 11 components as opposed to the instrument’s original 17 constructs [37].

The strength and unique ability of the PCPI-S instrument is that it addresses key areas of interest, including in depth exploration of relationship of ‘person’ with their own self and others, as well as work culture, and is based on an established theoretical framework.

### Relationship between domains of the person-centred practice framework

The Person-centred Practice Framework comprises of multi-layer domains measured through various constructs and its interplay. For example, the model proposes that the constructs on the outer layer influence the subsequent inner layer. As such, it is important to understand and examine the structural relationship between these constructs. While various studies have explored factors influencing person-centred practice, there is a gap in understanding the relationship between the factors, and whether there is any interplay between these factors and constructs. Understanding and establishing such relationship offers added value in guiding the transformation of person-centred practice by defining areas for prioritisation. It helps relevant policymakers, stakeholders and organisations to strengthen person-centred practices in a practical and optimised way. To address this gap, the study aimed to explore multidimensional and relationship pathways of person-centred care among primary healthcare providers. To achieve this, we will evaluate the measurement model through confirmatory factor analysis. We will then examine the roles of prerequisite of healthcare providers and care environment on how they affect delivery of person-centred processes, followed by the mediating effect of care environment towards the relationship between prerequisite and care processes.

## Methods

The domains based on the Person-centred Practice Framework were investigated through the hypotheses that test the relationships between the constructs. Structural equation modelling (SEM) was applied to identify the relationships through a path diagram. SEM is a multivariate statistical analysis that is increasingly used in healthcare. It allows assessment of reliability and validity of multi-items constructs measures [38, 39], followed by analyses of structural relationship between measured variables and latent constructs by combining factor analysis and multiple regression analysis. Additionally, the variance explained in the dependent variables accounts for both direct and indirect effects; hence it is larger with SEM than in multiple regression [40]. The protocol describing the study methods and the first part of the instrument’s validation are described elsewhere [37, 41].

### Participants & data collection

A survey utilizing PCPI-S instrument was conducted in primary healthcare clinics from three districts in a state of Malaysia over 3 months. Clinics with a daily patient load of more than 300 patients were invited to participate in the study. A minimum of 300 respondents were planned for analysis and validation based on the requirement of 5 to 10 respondents per item [42]. Responses were collected from healthcare providers from nine clinical categories: family medicine specialist, medical officer, pharmacist, medical assistant, nurses, occupational therapist, physiotherapist, dietitian and nutritionist. These participants recruited were healthcare providers who spend most of their work hours with service users in primary care clinics. We excluded any provider who was absent during the data collection period [42].

Prior to data collection, approvals were obtained from the state and districts authorities, followed by engagement sessions explaining the study objectives and data collection procedures to clinics representatives. The printed instrument was distributed to all eligible candidates, who were given 2 weeks to complete the answer. Completed instruments were returned in sealed envelopes to ensure confidentiality, while consent forms were collected separately.

### The operationalisation of person-centred practice’s theoretical framework

**Fig. 1:**
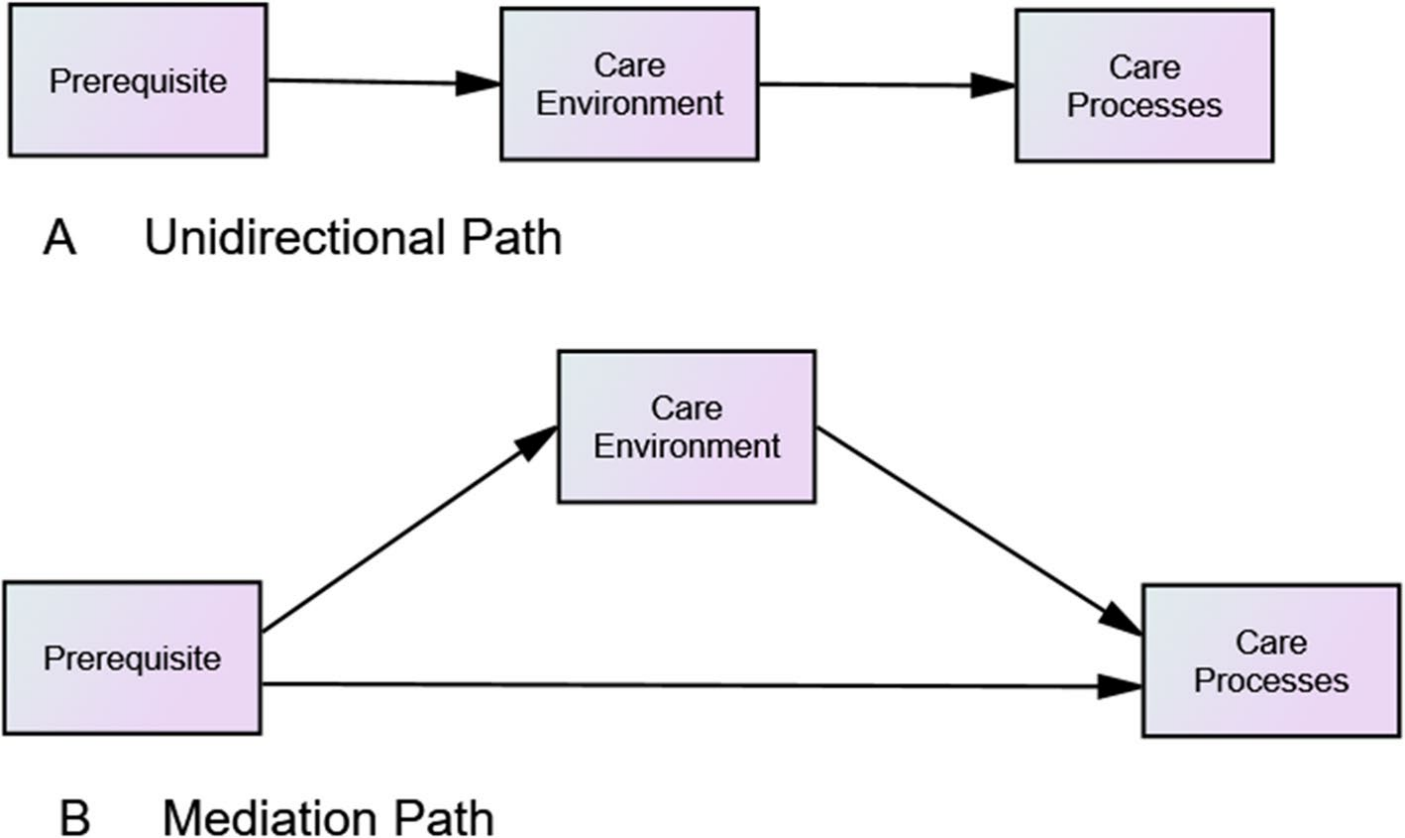
Pathways for operationalising Person-Centred Practice’s theoretical framework

The hypothesis assumes structural relationships between prerequisites, care environment and person-centred processes (henceforth simplified and renamed as care processes), as illustrated in the following path diagram (Fig. 1). In the unidirectional pathway (A), prerequisite among health care providers (Prerequisite) promotes formation of person-centred working environment (Care Environment) which then promotes delivery of person-centred care processes (Care Processes). In the mediation path (B), the following hypotheses was constructed:

1. prerequisite and care environment affect delivery of care processes; and
2. care environment mediates the relationship between prerequisite and care processes.

### Data analysis

A two-step approach was employed to test the relationship between constructs and variables. In step 1, confirmatory factor analysis (CFA) measured the reliability and validity of the model through measurements of convergent validity, discriminant validity and construct validity. Convergent validity was assessed based on Average Variance Extracted (AVE) and should exceed 0.5 to indicate how well the latent variables were reflected by the observed variables [43]. Discriminant validity was said to be achieved when the value of square root of AVE was higher than the values of correlation between constructs. Concurrently, the values of correlation between constructs should not exceed 0.85 [44, 45].

Based on the fitness indexes, factor loadings of observed variables forming latent constructs were inspected. Reliability was then assessed through the Composite Reliability (CR), which replaced Cronbach Alpha in measuring Internal Reliability using SEM [46].

After the measurement model was validated, SEM was employed to measure the goodness-of-fit of the developed model and examine the causal relationship between constructs and domains. The goodness-of-fit model was assessed through several fitness indexes from each category of three model fits: absolute fit, incremental fit and parsimonious fit. In this study, the fitness indexes used were Tucker-Lewis Index (TFI) (acceptable fit ≥0.9), Comparative Fit Index (CFI) (acceptable fit ≥0.9), Root Mean Square Error of Approximation (RMSEA) (acceptable fit ≤0.08), and Chi Square/degrees of freedom (acceptable fit ≤3.0) [43]. Bootstrapping procedure was applied to examine the mediation effect of care environment. Statistical analysis was performed using IBM SPSS AMOS (SPSS Inc) version 25 (IBM Corp, Armonyx).

## Results

### Respondents’ profile

A total of 919 respondents out of 1133 eligible candidates who fulfilled the inclusion criteria completed the instrument (81.1% response rate). Data with extreme outliers were excluded from analysis. Following this, 690 (75%) responses were analysed while maintaining the sample size and statistical power requirements. Respondents were mostly from the clinics of patient load more than 800 patients per day (*n* = 406). Half of the respondents were nurses (51%) which included the community nurse, registered nurse, sister and matron. Mean service years was 9.0 ± 6.7 (0.1–34 years). Table 2 shows the characteristics of clinics and respondents involved in the study.

**Table 2:** Clinics and respondents characteristics

Category	(***N*** = 690 respondents)
**Health profession**	**n (%)**
Family Medicine Specialist	8 (1.2)
Medical officer	142 (20.6)
Pharmacist	65 (9.4)
Nurse	354 (51.3)
Medical assistant	51 (7.4)
Physiotherapist	7 (1.0)
Occupational therapist	5 (0.7)
Nutritionist & Dietitian	8 (1.2)
Others (lab technician, assistant pharmacist)	6 (0.9)
Unknown	44 (6.4)
**Clinic type (patient attendance/day)**	**n (%)**
Type 1 (> 800)	406 (58.8)
Type II (500–799)	147 (21.3)
Type III (300–499)	137 (19.9)

### Measurement model

**Fig. 2:**
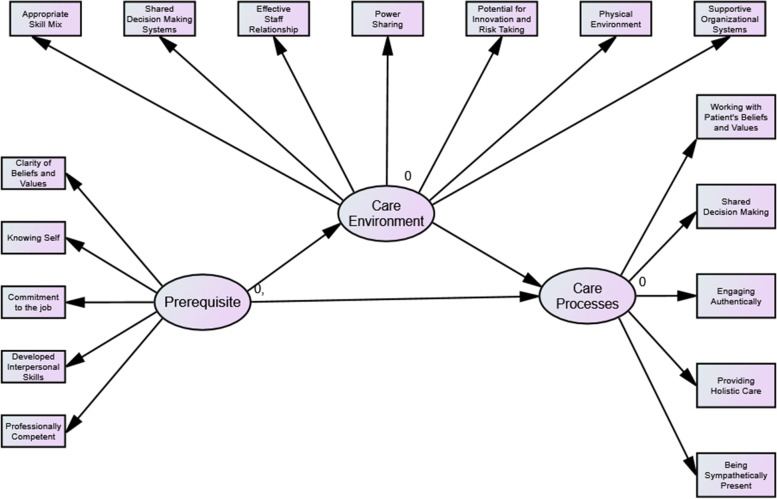
The framework model of the study

Figure 2 shows the framework of the original model which specifies the relationship between the domains. These 3 domains are second order constructs with certain number of sub-constructs where each sub-construct is measured using certain number of items.

Nine items were found with factor loadings < 0.6 (Table 3). Although removal of item with poor factor loading (< 0.6) from the model was suggested by literature as poor factor loading implied the item was less important in measuring the construct [43, 47], the items were retained in the overall instrument as they were crucial items in shaping person-centred care and contributed to the model fit. Dropping the items would render loss of opportunity to discuss important areas for improvement, therefore items were retained.

**Table 3:** Items with factor loadings < 0.6

Items	Factor Loading
A1: I have the necessary skills to negotiate care options	0.56
E16: I actively seek feedback from others about my practice	0.57
E17: I challenge colleagues when their practice is inconsistent with our team’s shared values and beliefs	0.36
F19: I recognise when there is a deficit in knowledge and skills in the team and its impact on care delivery	0.57
H28: My colleagues positively role model the development of effective relationships	0.49
I29: The contribution of colleagues is recognised and acknowledged	0.53
J34: I am able to balance the use of evidence with taking risks if needed	0.55
J35: I am committed to enhancing care by challenging practice	0.58
K37: I challenge others to consider how different elements of the physical environment impact on person-centredness	0.59

The values of Composite Reliability (CR) and Average Variance Extracted (AVE) for each domain are shown in Table 4, and the same values by sub-construct are demonstrated in Table 5. While the CR was generally high for all domains, many sub-constructs were found to have AVE values below 0.5. Some literature suggested convergent validity are adequate if AVE values are above 0.4, and accompanied by acceptable high values of CR, which are demonstrated by many subconstructs [48, 49], except for Professionally Competent, Clarity of Beliefs and Values, Appropriate Skill Mix, Power Sharing, and Innovation & Potential for Innovation and Risk Taking which have low AVE values.

**Table 4:** The Validity and Reliability of the measurement model by domains

Domain	Sub-construct	Factor Loading	CR^**a**^	AVE^**b**^
Prerequisite	Professionally Competent	0.86	0.85	0.60
	Developed Interpersonal Skills	0.79		
	Commitment to the job	0.94		
	Knowing Self	−0.02		
	Clarity of Beliefs and Values	0.86		
Care Environment	Appropriate Skill Mix	0.94	0.94	0.70
	Shared Decision-Making Systems	0.84		
	Effective Staff Relationship	0.73		
	Power Sharing	0.90		
	Potential for Innovation and Risk Taking	0.92		
	Physical Environment	0.88		
	Supportive Organisational Systems	0.61		
Care Processes	Working with Patient’s Beliefs and Values	0.90	0.95	0.80
	Shared Decision Making	0.90		
	Engaging Authentically	0.90		
	Providing Holistic Care	0.92		
	Being sympathetically present	0.84		

^a^ Cut off values ≥0.6

^b^ Cut off values ≥0.5

**Table 5:** Hypothesis testing

**Domain**	**Path**	**Domain**	**Estimate**	**S.E.**	**C.R.**	**P**	**Result**
Care Environment	<−--	Prerequisite	0.826	0.067	9.394	< 0.001	Significant
Care Processes	<−--	Care Environment	0.785	0.098	10.413	< 0.001	Significant

The skewness and kurtosis were not significant issues for all sub-constructs. The Discriminant Validity Index Summary presented in Table 6 showed the square root of each domain’s AVE exceeds its correlation value with other domains in the model, confirming that the discriminant validity for all constructs was achieved.

**Table 6:** The Discriminant Validity Index Summary

	Prerequisite	Care Environment	Care Processes
Prerequisite	**0.79**		
Care Environment	0.76	**0.80**	
Care Processes	0.76	0.75	**0.90**

### Structural model

Table 7 shows the path coefficient of the unidirectional pathway which states that person-centred prerequisite among health care providers promotes formation of person-centred care environment, which in turn promotes delivery of person-centred care processes. The relationship between Prerequisite and Care Environment was supported (β = 0.826, *p* < 0.001), as well as the relationship between Care Environment and Care Processes (β = 0.785, *p* < 0.001), confirming the model hypotheses.

**Table 7:** The Validity and Reliability of the measurement model by sub-constructs

Domain	Sub-Construct	Item	Factor Loading	CR^**a**^	AVE^**b**^
Prerequisite	Professionally Competent	A1	0.56	0.62	0.36
		A2	0.62		
		A3	0.61		
	Developed Interpersonal Skills	B4	0.66	0.77	0.45
		B5	0.65		
		B6	0.73		
		B7	0.65		
	Commitment to the job	C8	0.62	0.78	0.41
		C9	0.64		
		C10	0.62		
		C11	0.65		
		C12	0.68		
	Knowing Self	D13	0.71	0.77	0.53
		D14	0.83		
		D15	0.63		
	Clarity of Beliefs and Values	E16	0.57	0.52	0.28
		E17	0.36		
		E18	0.61		
Care Environment	Appropriate Skill Mix	F19	0.57	0.62	0.36
		F20	0.62		
		F21	0.60		
	Shared Decision-Making Systems	G22	0.69	0.82	0.53
		G23	0.77		
		G24	0.78		
		G25	0.68		
	Effective Staff Relationship	H26	0.82	0.76	0.52
		H27	0.81		
		H28	0.49		
		I29	0.53	0.72	0.39
	Power Sharing	I30	0.70		
		I31	0.62		
		I32	0.65		
	Potential for Innovation and Risk Taking	J33	0.67	0.63	0.36
		J34	0.55		
		J35	0.58		
	Physical Environment	K36	0.67	0.66	0.40
		K37	0.59		
		K38	0.63		
	Supportive Organisational Systems	L39	0.65	0.86	0.55
		L40	0.72		
		L41	0.77		
		L42	0.80		
		L43	0.76		
Care Processes	Working with Patient’s Beliefs and Values	M44	0.63	0.81	0.53
		M45	0.71		
		M46	0.75		
		M47	0.80		
	Shared Decision Making	N48	0.72	0.77	0.53
		N49	0.79		
		N50	0.66		
	Engaging Authentically	O51	0.75	0.80	0.57
		O52	0.72		
		O53	0.79		
	Providing Holistic Care	P54	0.70	0.76	0.51
		P55	0.74		
		P56	0.71		
	Being sympathetically present	Q57	0.75	0.85	0.66
		Q58	0.84		
		Q59	0.84		

^a^ Cut off values ≥0.6

^b^ Cut off values ≥0.5

**Fig. 3:**
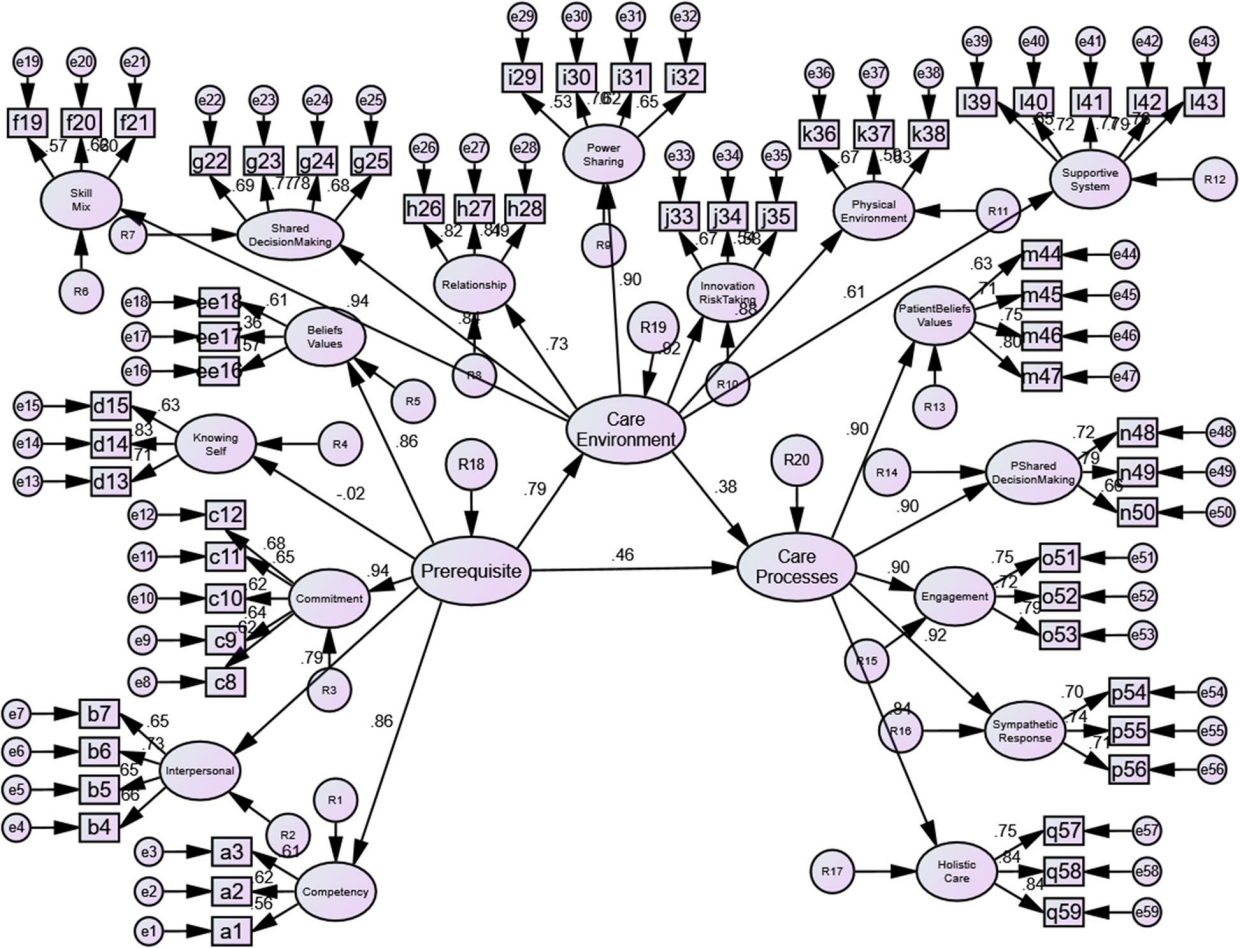
Final structural model

A single final model constituting all constructs was established to test the proposed hypotheses (Fig. 3), due to the structure of the original framework whereby each construct influenced one another. Modifications by allowing correlation errors between items to reach the final model were allowed (Additional file). The overall goodness-of-fit of the final model were all acceptable with RMSEA = 0.041, CFI = 0.903, TLI = 0.895 and ChiSq/df = 2.158.

### The mediation analysis

Maximum likelihood bootstrapping procedure with bootstrap sample of 1000 and bias correction confidence interval of 95% was performed. Based on suggested calculation by Zainudin [46], it was found that the direct effect between Prerequisite and Care Processes (0.64) was bigger than the indirect effect mediated by Care Environment (0.542 X 0.827 = 0.45). However, the positive pathways were nevertheless present and significant, as shown in Table 8, therefore establishing Care Environment as a partial mediator between Prerequisite and Care Processes.

**Table 8:** Bootstrap procedure in testing Care Environment as a mediator on the relationship between Prerequisite (P) and Care Processes (CP)

	Indirect Effect P - CP	Direct Effect P - CP
Bootstrapping results	0.45	0.640
Bootstrapping *p*-value	0.002	0.002
Result	Significant	Significant
Type of mediation	Partial Mediation since both direct and indirect relationships were significant	

## Discussion

Overall, the validity and reliability of the instrument were acceptable. However, there were some low AVE across a few sub-constructs, suggesting suboptimal convergent validity. This is most likely due to differing interpretation of person-centred practice among Malaysian healthcare providers, influencing the factoring of items, as shown and discussed in the first part of the instrument’s validation [37]. It should be noted that no items were dropped due to its significant contribution to the overall model. These results are in line with previous studies in Norway and Germany where six and four items not achieving acceptable factor loading value were all retained [35, 36]. The model goodness-of-fit were positive, in concordance with the previous studies confirming the model fit [34–36]. Correlation of errors were allowed in the final model - suggesting the possibility of item misinterpretation, given the influence of culture, context, language and local practice, therefore affecting the relationship between items and their latent constructs, as shown previously [37].

The overall model substantiated the order of the Person-centred Practice Framework. Indeed, significant relationship was found in strengthening person-centred prerequisite to establish a person-centred working environment and finally promote delivery of person-centred processes. Previous studies have also shown importance of healthcare providers’ attributes and work culture in improving healthcare delivery and quality of care [50–53]. When work culture is supported, healthcare providers’ motivation to work increased and level of stress reduced [54]. Presence of excellent teamwork was also an indicator of staff’s intrinsic motivation and implied a high-functioning organisation in terms of communication, support and meeting staff expectations [50–52].

The provision of a person-centred practice and formulation of a supportive work culture, however, is not without challenges. Manley suggested work culture to be the culture at workplace that service users, residents, and staff experience every day. It extends beyond ‘organisational artefacts’ of visible products such as language, technology and forms of address, and covers values such as shared ideas, effective teamwork, continuous learning environment, and transformational leadership, among others [55]. McCormack et al. described what healthcare providers usually experienced was mere ‘person-centred moments’, that is when things seem to come together and was rewarding, when in reality it depends solely on good traits of individuals and leaders, hence rarely repeatable on a daily basis [7]. It is therefore crucial to understand that person-centred care is not bound, or measured by the quality of health service received by service users, neither by their health outcomes alone, rather by how persons are viewed and treated while receiving care. Moreover, the values of mutuality, collegiality, and care are not necessarily transformed into practice despite being embedded in the mission statements and organizational frameworks. Similarly, team work and team effectiveness were always thought to be commonly in place, but dysfunctional team relationships and discrepancy between envisioned and practiced healthcare continue to exist [56, 57].

This finding emphasises the importance of establishing a supportive and effective working environment and work culture in promoting delivery of person-centred processes, whereby it does not only need to be present to allow the process to happen, it also need to be in place in mediating the person-centred values held by healthcare providers. Developing person-centred cultures therefore need persistent effort and commitment from all parties involved [27].

While the Person-centred Practice Framework outlines all the components and criteria that need to be present, how can they be transformed into practice? Studies utilising practice development methodology demonstrated how the transformation took place [27, 58]. In order to motivate staff to adopt PCC values and attributes, enabling factors must be in place. This includes individual factors such as having transformational leadership skills, offering skilled facilitations to staff, and clarifying roles of all staff. At organisational level, factors enabling the change include effective management, enabling approach to leadership and decision-making, organisational preparedness, and supportive human resource department [59]. Transformational programme proposed, based and prioritised on issues identified by staff, and tailored to the need of the staff and community could then be rolled out, over a period deemed required for the changes to happen. Along the process, evaluation and feedback should be gathered to improve implementation and resolve conflicts [27, 58, 59].

One of the major strengths of the study was exploration of person-centred concepts in a theoretical framework based on established theories. It helps strengthen existing knowledge in the wide PCC field. To our knowledge, this was the first study that investigated the relationship among all domains of PCPI-S questionnaire in a primary care context, extending the instrument applicability into a wider healthcare setting. Nonetheless, since the study only focused on healthcare providers working in public primary care settings, the findings were heavily influenced by the work culture of this setting, and might not necessarily be applicable to a different setting. The model should be validated in multiple samples from various healthcare settings in future studies. Since this study was cross-sectional in nature, the direction of causality for some factors could not be established. Future studies should adopt a longitudinal research design to prove causality. As with all structural models, one disadvantage of using SEM is that the model represents approximations that may omit variables implicated in causal processes or other features [60]. Therefore, future studies are encouraged to investigate the inclusion of other relevant variables in the model.

## Conclusions

The findings of this work support the original framework in establishing the pathway that show person-centred prerequisite is needed to form a person-centred care environment, which then needed to allow delivery of person-centred care processes. It is shown that a person-centred care environment also acts as partial mediator between prerequisite and care processes. Therefore, in order to successfully move towards delivering person-centred practice, it is imperative to equip our healthcare providers with person-centred values and beliefs, while at the same time transform current work culture to align with person-centred care.

## Supplementary Information

**Additional file 1.** Correlation of errors allowed in the final structural model.

## Data Availability

The dataset that supports the findings of this article belong to the Primary Care Systems for Person-Centred Provider Practices study. Requests for the data can be obtained from Dr. Mohd Azahadi Omar (drazahadi@moh.gov.my), the head of sector for Biostatistics & Data Repository, National Institute of Health, Ministry of Health Malaysia, with the permission from the Director-General of Health, Malaysia.

## Abbreviations

PCC: Person-Centred Care
PCPI-S: Person-centred Practice Inventory-Staff
SEM: Structural Equation Modelling
CFA: Confirmatory Factor Analysis
AVE: Average Variance Extracted
CR: Composite Reliability
TFI: Tucker-Lewis Index
CFI: Comparative Fit Index
RMSEA: Root Mean Square Error of Approximation
