# Supplementary material for: Healthcare provider person-centred practice: relationships between prerequisites, care environment and care processes using structural equation modelling
Source: BMC Health Serv Res. 2022 Apr 29;22:576. doi: 10.1186/s12913-022-07917-3 (PMC9052661; doi:10.1186/s12913-022-07917-3)
Supplement: Supplementary file 1 — Additional file 1. Correlation of errors allowed in the final structural model. [file 12913_2022_7917_MOESM1_ESM.docx]

# **Additional file**

Correlation of errors allowed in the final structural model

| Correlated errors | Modification Index |
| --- | --- |
| h28 - i29 | 171.5 |
| j33 - i32 | 119.9 |
| p56 - q57 | 68.7 |
| ee17 - f19 | 54.5 |
| g22 - f21 | 54.5 |
| m44 - l43 | 48.1 |
| h28 - i30 | 40.2 |
| m44 - Supportive system | 39.6 |
| f21 - i29 | 35.9 |
| h26 - g25 | 33.7 |
| ee18 - i29 | 32.6 |
| b5 - i29 | 29.9 |
| k38 - l39 | 26.3 |
| j33 - l40 | 27.8 |
| n50 - o51 | 27.7 |
| i31 - i29 | 25.9 |
| h28 - i31 | 26.8 |
| f21 - o53 | 25 |
| ee17 - k37 | 23.3 |
| m44 - a1 | 23.6 |
| l41 - l40 | 23.1 |
| l39 - l40 | 26.7 |
| h27 - k38 | 22.8 |
| k36 - p56 | 22.1 |
| ee18 - f21 | 21.4 |
| g22 - ee18 | 20.5 |
| m44 - i30 | 21 |
| l39 - i29 | 20.4 |
| j35 - h26 | 23.4 |
| f21 - o51 | 21 |
| f20 - ee17 | 20 |
| f19 - h28 | 20.4 |
| l39 - h28 | 19.2 |
| p56 - q59 | 19.5 |
| k38 - l40 | 18 |
| i31 - j33 | 19 |
| k36 - n50 | 18.3 |
| g24 - h26 | 17.6 |
| f21 - h28 | 17 |
| c8 - b5 | 16.8 |
| ee17 - b5 | 15.4 |
| h27 - j35 | 17.2 |
| f21 - b5 | 15.1 |
| m45 - m44 | 15.3 |
| n50 - f21 | 15.1 |
| m44 - h27 | 15.7 |
| k38 - c10 | 15.4 |
| c9 - c10 | 15.9 |
| h28 - m46 | 14.6 |
| h28 - q59 | 14.4 |
| p56 - q58 | 13.9 |
| m44 - k37 | 14.4 |
| m44 - c11 | 15.5 |
| ee18 - f20 | 13 |
